# The Tumor Vascular Endothelium as Decision Maker in Cancer Therapy

**DOI:** 10.3389/fonc.2018.00367

**Published:** 2018-09-10

**Authors:** Diana Klein

**Affiliations:** Institute of Cell Biology (Cancer Research), University Hospital, University of Duisburg-Essen, Essen, Germany

**Keywords:** neovascularization, angiogenesis, radiotherapy, anti-angiogenic therapy, vascular stabilization, immune escape

## Abstract

Genetic and pathophysiologic criteria prearrange the uncontrolled growth of neoplastic cells that in turn initiates new vessel formation, which is prerequisite for further tumor growth and progression. This first endothelial lining is patchy, disordered in structure and thus, angiogenic tumor vessels were proven to be functionally inferior. As a result, tumors were characterized by areas with an apparent oversupply in addition to areas with an undersupply of vessels, which complicates an efficient administration of intravenous drugs in cancer therapy and might even lower the response e.g. of radiotherapy (RT) because of the inefficient oxygen supply. In addition to the vascular dysfunction, tumor blood vessels contribute to the tumor escape from immunity by the lack of response to inflammatory activation (endothelial anergy) and by repression of leukocyte adhesion molecule expression. However, tumor vessels can remodel by the association with and integration of pericytes and smooth muscle cells which stabilize these immature vessels resulting in normalization of the vascular structures. This normalization of the tumor vascular bed could improve the efficiency of previously established therapeutic approaches, such as chemo- or radiotherapy by a more homogenous drug and oxygen distribution, and/or by overcoming endothelial anergy. This review highlights the current investigations that take advantage of a proper vascular function for improving cancer therapy with a special focus on the endothelial-immune system interplay.

## Introduction

New vessel formation is a hallmark of tumor growth and progression (1–3). Once a critical tumor mass (of approximately 1–2 mm^3^) has formed, the metabolic demands of the growing cancer cells together with the diffusion limits of nutrients and oxygen foster the generation of a tumor-associated neovasculature (4). Known as the angiogenic switch, this process is regulated directly and indirectly by the tumor using a variety of pro- and anti-angiogenic signaling molecules, including vascular endothelial growth factor (VEGF), platelet-derived growth factor (PDGF), angiopoietins and thrombospondins, among others (5, 6).

**Figure 1:**
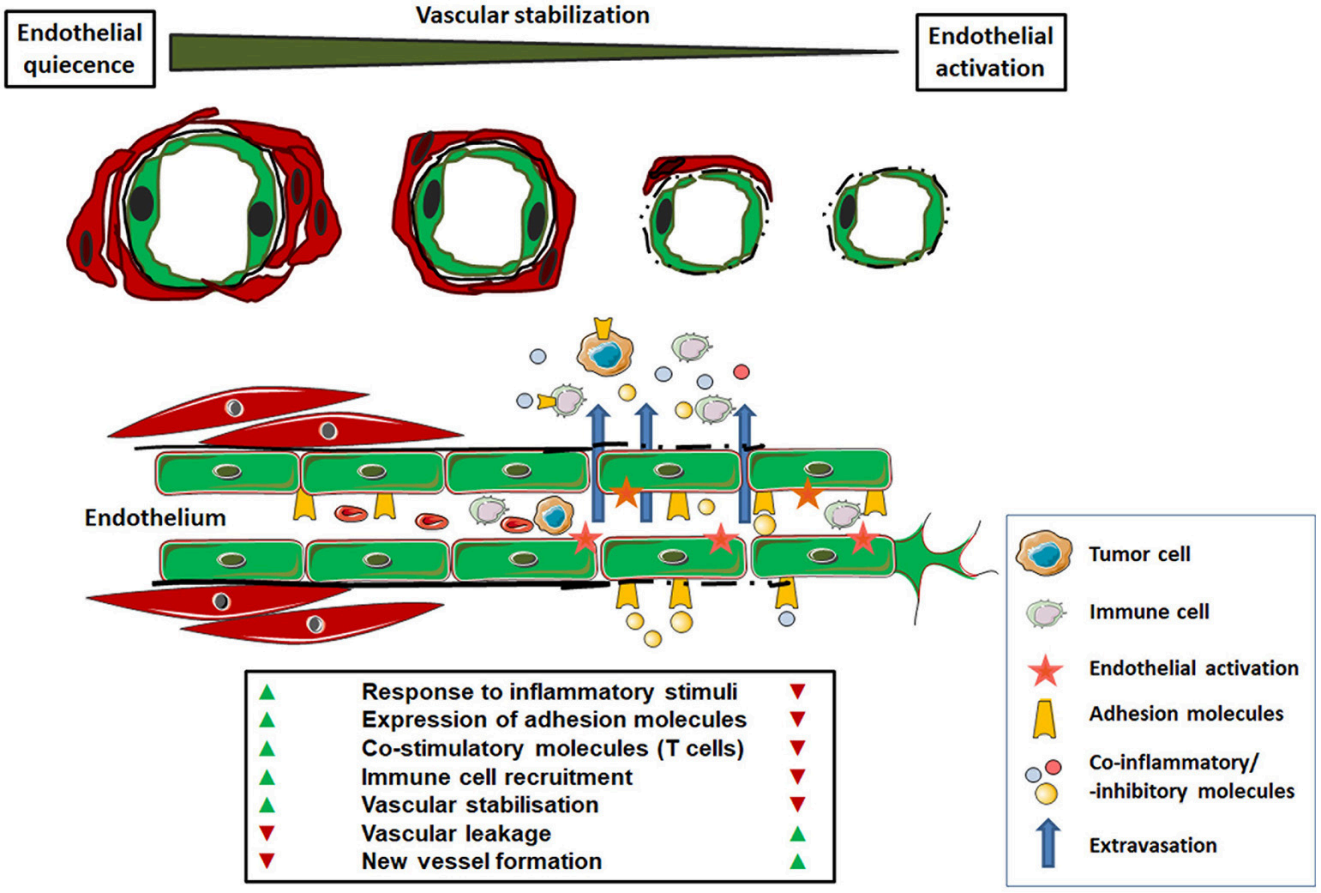
Functional characteristics of normal versus tumor-associated endothelium. In the healthy state, mature endothelial cells (shown as green cells) are characterized by quiescence. A regular blood flow and pressure is achieved by vascular stabilization, which is the association and integration of vascular mural cells (smooth muscle cells and pericytes; shown as red cells). Thus, the normal endothelium provides an efficient barrier to liquids or cell extravasation. Upon activation, e.g., in response to inflammatory signals, normal endothelial cells can up-regulate cell adhesion molecules (selectins and integrins) for the capture, rolling and arresting of circulating immune cells prior tissue extravasation. The anergic tumor endothelium lacks that response to inflammatory stimuli. In response to tumor-secreted angiogenic factors the endothelium becomes activated. This activated and/or “angiogenic” endothelium phenotype is characterized by a missing or defective basement membrane and structural instability (lack of vascular mural cells), which leads to increased vascular leakage. In addition, these newly formed and functional abnormal blood vessels are chaotically organized which, together with endothelial anergy, limits the effective immune cell distribution and tissue infiltration. The altered expression of co-stimulatory and–inhibitory molecules with the potential to block anti-tumor immune cells further contributes to an immunosuppressive microenvironment within the tumor.

In contrast to the normal, usually quiescent vasculature, tumor blood vessels were proved to be functionally abnormal because of their immature phenotype: the endothelial lining is patchy, the basement membrane is defective or discontinuous and respective vessel walls lack the mural elements (smooth muscle cells and pericytes); so they cannot actively respond to physiologic stimuli (Figure 1) (7, 8). Thus, there is relative imbalance between tumor tissue and the formation of adequate vascular structures, which finally results in tumor areas with an apparent oversupply in addition to areas with an undersupply of vessels. This complicates not only the efficient distribution of nutritions and oxygen but also the effective administration of cancer therapeutics. Even at the molecular level, i.e., regarding the expression of important signaling molecules, receptors or cell adhesion molecules in the tumor vascular bed, there is an imbalanced state between pro- and anti-oxidants, -inflammatory molecules, and -coagulation signals (9–11). As a result, tumor endothelial cells bear immune-regulatory properties: alterations in the immune cell attraction and activation, as well as in the expression of co-stimulatory and -inhibitory molecules can promote immune tolerance and thus generate an immune-privileged tumor microenvironment (12–14).

**Figure 2:**
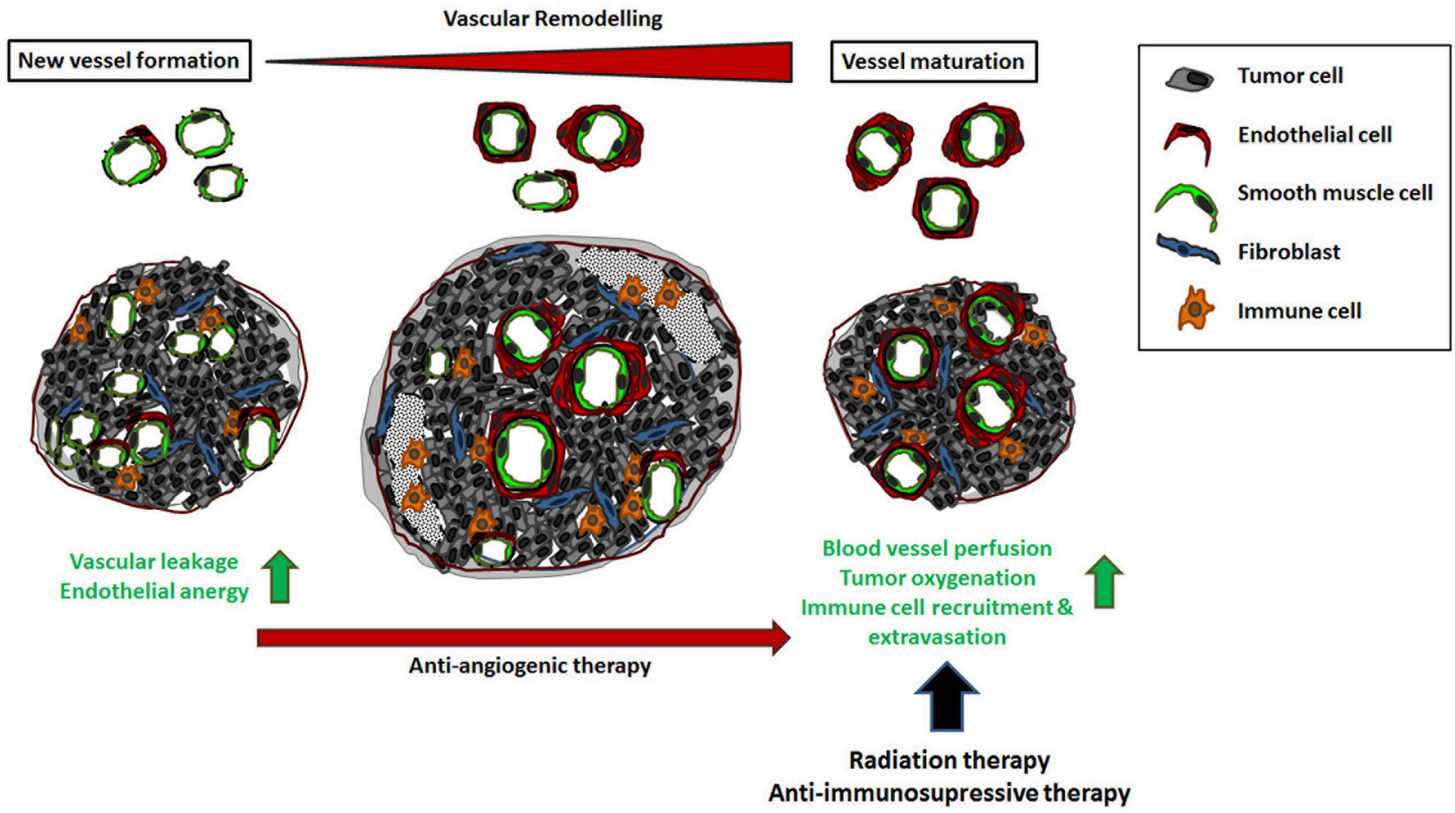
The impact of vascular remodeling for cancer therapy. Tumor neovascularization supplies a high dense network of chaotically organized, immature and unstable vessels. Vascular dysfunction as well as the unresponsiveness to inflammatory stimuli results in an uneven blood flow and pressure as well as an inefficient distribution of blood stream components, including circulating immune cells. This complicates the effective administration of cancer therapeutics. In the course of tumor progression, these angiogenic vessels can mature by the association of vascular mural cells (vascular remodeling) that stabilizes the immature vessels resulting in normalization of the vascular structures. Vascular remodeling is dynamic and strictly regulated process; an ordered remodeling seems to be critical for proper vascular development, maintenance and stability of the vessel wall. The process of vascular stabilization is accelerated in cancer therapy when anti-angiogenic agents were applied. As a result, blood vessel perfusion and thus oxygenation as well as the efficient distribution of applied drugs are improved. In addition, vascular maturation and normalization restores the potential of the tumor endothelium to recruit and direct circulating immune cells to the tumor tissue.

However the newly formed tumor vessels can remodel in terms of vascular maturation within the course of tumor progression (7, 10, 15, 16). Herein, a partial stabilization, which is achieved by the association and integration of vascular mural cells occurs particularly in the central areas of the tumors, which is associated with a significant reduction of vascular densities and augmented necrosis in these tumor regions (Figure 2) (7, 16, 17). The process of vascular remodeling within a tumor is influenced by the cancer therapy. Especially in anti-angiogenic therapy, angiogenesis inhibitors foster vascular stabilization and a partial normalization of the tumor vascular bed, which is supposed to improve the efficiency of the previously established therapeutic approaches, such as chemo-, radio-, and/or immuno-therapy (18–20). This review highlights the central role played by the tumor vascular endothelium for cancer therapy and summarizes the current strategies that take advantage of a proper vascular function for overcoming anti-tumor immunity and thus improving immunotherapy.

## Endothelial activation and dysfunction

One important physiological function of normal endothelial cells is quiescence of the inflammatory response and thus, participation in immune surveillance (21, 22). Quiescent endothelial cells fail to provide the requisite signals for leukocyte recruitment; but the cells can be activated to express adhesion molecules and to release chemokines that promote capture and transmigration of blood leukocytes into tissues. Endothelial cell activation can typically induced by multiple factors, including circulating inflammatory cytokines, such as tumor necrosis factors (TNF) and interleukins (IL), reactive oxygen species, oxidized low density lipoprotein, autoantibodies and traditional risk factors directly and indirectly activate endothelial cells (21). The term activated endothelium implies a change in endothelial cell morphology (23). Endothelial activation was further specified as a change in surface molecules and in endothelial cell functions in response to cytokine treatment, and it was emphasized that these changes does not represent endothelial cell injury or dysfunction (24, 25). Components of endothelial cell activation are upregulation of surface antigens (e.g., HLA molecules) and leucocyte adhesion molecules (e.g., E-selectin, ICAM-1/2, and VCAM-1), pro-thrombotic endothelial cell changes (e.g., loss of the surface anticoagulant molecules thrombomodulin and heparan sulfate), cytokine production (e.g., IL6, IL8, MCP1), and changes in the vascular tone (e.g., loss of vascular integrity, expression of vasodilators, and NO). These components mutually interact in causing local inflammation (25). Endothelial activation also leads to an increase in angiopoietin-2, which is known to destabilize barrier function and promote inflammation (26). The recruited and extravasated immune cells appear then in vicinity of the activated endothelial cells, and can further become activated (23). Importantly, the phenotype of activated endothelial cell is reversible and can return to the quiescent, non-activated phenotype when the activating factors were removed (27–30). Prolonged activation of the endothelium can be associated with the loss of microvascular barrier integrity and subsequent vascular injury or progress to endothelial cell apoptosis (31).

## The tumor endothelium

Phenotypic differences at the molecular and functional levels have been identified for tumor and normal endothelial cells (32). Tumor secreted growth factors, and in particular VEGF, are the principal drivers of most the fundamental morphogenetic events involved in the induction of tumor vascularization including activation of the hitherto quiescent endothelium in terms of stimulating endothelial cell proliferation and migration (33). Many tumor types are characterized by a VEGF upregulation. Tumor hypoxia can also foster increased VEGF expression levels, which in turn perpetuates angiogenic processes (34). Tumor endothelial cell are very heterogeneous and thus vascular function of respective tumor blood vessels vary depending on the type of tumor and progression stage (35, 36). The newly formed blood vessels of tumors as well as of metastatic tumors are more immature with fewer pericytes. In general, tumor endothelial cells are characterized by a proangiogenic phenotype, with the upregulation of several angiogenesis-related genes, such as VEGFR1/R2 and matrix metalloproteinases (MMPs) to modulate the basement membrane and degrade the extracellular matrix allowing endothelial cell migration. The resulting tumor vascular bed is disorganized, tortuous, and the leaky phenotype of angiogenic tumor blood vessels that is accompanied by an irregular blood and heterogeneous permeability limits for the efficient distribution of blood components within the tumor mass. Further on, the structural abnormalities like poorly interconnected endothelial cells, no regular associated mural cells, and abundance of vesiculo-vacuolar organelles contribute to the leaky, hyper-permeable phenotype, finally causing extravasation of intravascular fluids and plasma proteins (37, 38). Therefore, an markedly increase in the intra-tumor fluid pressure throughout the tumor is observed, while normal pressure values were found in the tumor's periphery or in the surrounding tissue (39, 40). The high tissue pressure within the tumor, together with mechanical stress from the proliferating cancer cells and the extra mass of generated matrix, is able to collapse tumor vessels, that means closing their lumen through compressive forces, leading to the collapse of the blood vessels and finally resulting in hypoxia (32, 38, 41). The compromised blood flow in tumor blood vessels further decreases oxygen and nutrient supply, causing physiological stress to the tumor. The physiological microenvironments of many macroscopic tumors were therefore characterized by high interstitial fluid pressure (interstitial hypertension), which besides nutrient deprivation and hypoxia in turn was associated with malignant progression, development of metastatic disease and a poor disease-free survival in a large number of cancer types (42–44).

Angiogenic growth factors were further shown to suppress the expression of adhesion molecules involved in leukocyte binding (e.g., ICAM-1/2, VCAM-1, E-selectin and CD34) in tumor endothelial cells, which then causes the unresponsiveness of tumor endothelial cells to inflammatory signals, a phenomenon called endothelial cell anergy that causes lymphocyte tolerance (45–48). Hence, the interaction of leukocytes with the endothelial cells lining the vessels is reduced, and thus intra-tumoral recruitment of effector T-cells, either induced or adoptively transferred, is impaired and subsequently fail to exert the anti-tumor effects necessary to eradicate the tumor (49, 50). This is one of the mechanisms tumors have developed to escape the immune surveillance (51). Concerning the mechanism, angiogenic growth factor like VEGF and bFGF inhibited the TNF-mediated activation of NF-KB. In addition, bFGF induced hyperphosphorylation of p38 MAPK on endothelial cells (52, 53). Promoter histone modifications were further shown to mediate tumor endothelial cell anergy, as adhesion molecule expression was shown to be epigenetically repressed in tumor endothelial cells, and that DNA methyltransferase and histone deacetylase inhibitors which have angiostatic activity could re-induce expression of the *ICAM-1* gene by reversal of histone modifications in the *ICAM-1* promoter, thereby restoring leukocyte-vessel wall interactions and leukocyte infiltration (51).

## Tumor endothelium-mediated regulation of the immune response: clinical implications for targeting the tumor vasculature to improve immunotherapy

A functional vascular network is prerequisite not only for nutrients or oxygen supply but also for the immune cells to enter the tissues. The functional and structural abnormalities of tumor blood vessels together with the unresponsiveness of the endothelium to inflammatory stimuli caused by proangiogenic factors decrease the recruitment of immune effector cells into the tumor, thus limiting the effectiveness of cancer immunotherapies (54, 55). Given that the abnormal tumor vasculature contributes to the immune-suppressive tumor microenvironment, processes of vascular normalization in terms of vessel maturation were supposed to potentiate cancer immunotherapy by promoting immune cell infiltration into tumors and reducing the immune suppression within the tumors (55, 56).

Today, immunotherapy for activating therapeutic anti-tumor immunity has become a mainstay of cancer therapy (57, 58). Although the use of monoclonal antibodies directed against cytotoxic T-lymphocyte associated protein 4 (CTLA-4) and the programmed cell death-1 (PD-1/CD279) T-cell receptor and/or its ligand (programmed death-ligand 1 (PD-L1/B7-H1/CD274) showed unprecedented durable responses in some patients with a variety of cancers, acquired resistance to immune checkpoint antibody blockades was commonly observed in most cancer patients (59, 60).

The different approaches being currently explored to increase recruitment of immune effector cells, include manipulating the expression of homing-associated molecules on T-cells and tumor endothelial cells. Concerning the first option, a successful approach to target or restore tumor-induced immunosuppression was made by adoptive cell therapy using tumor-reactive T-lymphocytes that resulted in objective tumor regression in >50% of treated patients (61). The potential to treat a wide range of solid cancers with autologous T-cells was further highlighted when re-directed T-cells expressing a non-MHC restricted chimeric antibody receptor (recognizing CD19 on B-cells) in refractory B-cell malignancies were successfully used to overcome dominant immunosuppression (62, 63). However, the success of such therapies again depends on applied agents (here the lymphocytes) in finding their desired place, leaving the bloodstream and subsequently infiltrating the tumor tissues (12, 64).

Thus, strategies addressing directly the vascular system to sensitize tumors or improve the therapeutic response in cancer therapy were established and already shown to exert beneficial effects in immune checkpoint blockade. In a very elegant preclinical study Elia et al. showed that a selective (pre)activation of the tumor endothelium with the cytokine TNF promoted intratumoral T-cell infiltration, and immune checkpoint blockade (65). The authors used low doses of NGR-TNF, a Cys-Asn-Gly-Arg-Cys peptide-TNF fusion product, in simultaneous combination with anti-CTLA-4 and anti-PD-1 antibodies to treat transgenic adenocarcinoma of the mouse prostate (TRAMP) mice with autochthonous prostate cancer and mice with orthotopic B16 melanoma. NGR-TNF administration was already used as a safe and therapeutic systemic administration to target TNF selectively to angiogenic tumor vessels which then altered the endothelial barrier function together with an upregulation of leukocyte-endothelial cell adhesion molecules, the release of pro-inflammatory cytokines, and the infiltration of tumor-specific effector CD8(+) T-cells. As a result, NGR-TNF enhanced the therapeutic activity of adoptive and active immunotherapy, delaying tumor growth and prolonging survival (66, 67). Finally, the combined therapy had beneficial effects on endogenous immune surveillance, through depletion of regulatory T-cells and expansion of a fully functional, polyclonal repertoire of cytotoxic T-lymphocytes (65).

Proper vascular function as revealed by measurements of vessel perfusion was further used to predict the therapeutic response to immune checkpoint blockade (68). Here, the authors used clinically relevant mouse breast tumor models that were either sensitive or resistant to immune checkpoint blockade treatment (with anti–CTLA4 and anti–PD1 agents) and thus mirror cancer progression and therapy response in humans. A significantly enhanced vessel perfusion was observed mostly in treatment-sensitive tumors, which was accompanied by an accumulation of CD8+ T-cells and interferon-gamma production, strongly suggested that increased vessel perfusion reflects the successful activation of anti-tumor T-cell immunity by immune checkpoint blockade (68). Thus, the authors reported here a reliable and noninvasive indicator for predicting immune checkpoint blockade responsiveness which was related to proper vascular function of the tumor endothelium.

Conclusively, tumor endothelial cells are actively involved in immune cell exclusion and inhibition of lymphocyte activation, fostering an immunosuppressive intratumoral microenvironment that contributes to the tumor immune escape and severely impairs conventional cancer therapies (9, 14, 69). Hypothetically, tumors resistant to immune checkpoint blockade could become sensitive to such treatment again when the tumor endothelium specific alterations in leukocyte-endothelial adhesive interactions were normalized. In line with this idea, Huang et al. showed that synchronizing vascular normalization by antiangiogenic (anti-VEGFR2) therapy with T-cell activation induced by a whole cancer cell vaccine therapy enhanced anticancer efficacy in a CD8(+) T-cell–dependent manner in both immune-tolerant (MCaP0008) and immunogenic (MMTV-PyVT) murine breast cancer models (56). Even the administration of an antibody against mouse VEGF synergized with adoptive cell transfer-based immunotherapies (70). Herein, normalization of the tumor vasculature through disruption of the VEGF/VEGFR-2 axis increased extravasation of adoptively transferred T-cells into the tumor. Combining VEGF blockade with an additional blockade of angiopoietin-2 by a bispecific antibody provided superior therapeutic benefits in the melanoma cancer as well as in metastatic breast and pancreatic cancer models (71, 72). Neutralization of both angiogenic factors resulted in vascular regression of angiogenic blood vessels whereas the remaining blood vessels were normalized and facilitated the extravasation and perivascular accumulation of activated, IFNγ-expressing CD8(+) cytotoxic T lymphocytes (72). The perivascular T-cells in turn induced the expression of PD-L1 in tumor endothelial cells via IFNγ, which was utilized when additionally PD-1 blockade improved tumor control by the bispecific antibody in the different cancer models.

Using regulator of G protein signaling 5-deficient mice, a genetically induced vascular normalization mouse model, in which newly formed blood vessels were characterized by a mature and thus stabilized phenotype, it was further shown that tumor vessel normalization consequently reduced vascular leakiness and hypoxia within the tumors, leading to an influx of immune effector T-cells (22, 30). Herein, vessel maturation was accompanied by a restoration of endothelial cell anergy as adhesion molecules on the luminal surface of tumor endothelial cells were increased and more uniformly distributed. Furthermore, the use of anti-angiogenic therapy was shown to normalize the tumor vasculature and thereby improve cancer immunotherapies.

Instead then of starving tumors from their blood supply and achieving complete vessel regression, vessel normalization by anti-angiogenic therapy has gained more attention for generating more mature and regular functioning tumor blood vessels with increased vessel perfusion. This is supposed to improve distribution of circulating blood components, oxygenation, removal of suppressive metabolites, as well as distribution of therapeutically applied drugs (56). In addition, anti-angiogenic therapy mediated vessel normalization was shown to reverse endothelial cell anergy resulting in (re)sensitizing tumor blood vessels to inflammatory stimuli by inducing homing molecule expression and thus an improved T-cell-dependent anti-cancer immunity (12, 70, 73).

Improving the aberrant structural abnormalities and associated dysfunctionalities of tumor blood vessels, and thus lowering tumor hypoxia and enabling immune cell infiltration, by antiangiogenic therapy was shown to synergize with immunotherapies for more durable effects (74). In an preclinical study using the polyoma middle T oncoprotein breast cancer and the Rip1-Tag2 pancreatic neuroendocrine tumor mouse models it was shown that anti-angiogenic therapy can improve anti-PD-L1 treatment and further, the other way round that anti-PD-L1 therapy can sensitize tumors to anti-angiogenic therapy and prolong its efficacy (74). Herein, vessel normalization (as shown by reduced microvessel densities, increased diameters and a regular pericyte coverage) promoted lymphocyte infiltration and enhanced cytotoxic T-cell activity.

In addition, to tumor endothelial cell anergy that limits the adhesion and subsequent extravasation of recruited leukocytes, tumor-derived factors can further induce endothelial cell-mediated apoptosis of recruited immune cells, e.g., by induced death mediator Fas ligand (FasL, also called CD95L) expression which directly kills anti-tumor T-cells finally leading to an inefficient recruitment of effector CD8(+) T-cells into the tumor (12, 75). Within the tumor endothelium of breast, prostate, colon, bladder, renal cancers a selective expression of FasL was reported that was associated with scarce CD8(+) infiltration and a predominance of FoxP3(+) regulatory T-cells (76). As the induced FasL expression in tumor endothelial cells which acquired the ability to kill effector CD8(+) T-cells but not regulatory T-cells was mediated by tumor-derived VEGF, IL10 and prostaglandin E2 cooperatively, the authors proposed a “tumor endothelial death barrier” that contributes to the tumors immune escape cells (76). The tumor endothelium was also shown to express increased levels of PD-L1 under inflammatory conditions, which in turn was able to bind to PD-1 on activated lymphocytes to negatively control T-cell activation (77–79).

Another molecule which became of interest for activating therapeutic anti-tumor immunity is the interferon-inducible intracellular enzyme indoleamine 2,3-dioxygenase 1 (IDO-1), which catalyzes the initial and rate-limiting step in the degradation pathway of the essential amino acid tryptophan to kynurenine (80, 81). Kynurenines in turn induces proliferation, activation and recruitment of T regulatory cells and myeloid-derived suppressor cells that further suppress tumoricidal T-cells. Increased IDO-1 expression levels were already associated with tumor progression, poor prognosis, and a decreased overall survival (82, 83). IDO-1 expression can be found in different tumor cells, normal epithelial cells, monocyte-derived cells and in particular also in tumor endothelial cells (84–86). Of note, in some tumor entities, the tumor endothelial cells rather than tumor cells were shown to be responsible for increased IDO expression, e.g., in metastatic renal cell carcinoma (84). IDO-1 expression levels in tumor endothelial cells were further suggested being a predictive biomarker for the response to immune-based cancer therapy (86–88). For example, in colorectal cancer, IDO-1 expression by host endothelial cells was a negative prognostic factor for regression free survival, independent of disease stage (89). Therefore, an inhibition of the (endothelial-specific) IDO-1 signaling pathway could be a promising novel adjuvant therapeutic strategy for clinical application in immunotherapy.

However, the actively participation of tumor endothelial cells in the innate and adaptive immune responses is not limited to the ability to attract and direct a wide range of immune cells and elevate extravasation from the host circulation. Tumor endothelial cells are believed to have a role in antigen presentation (9, 13, 14). Endothelial cells were found to act as antigen presenting cells by constitutively expressing major histocompatibility complex I and II molecules and presenting endothelial antigens to T-cells resulting in T-cell activation (90, 91). Endothelial cells also were shown to express the co-stimulatory molecules CD80 and CD86 that are essential for activation of naïve T-cells, but following transplantation only activation of CD4(+) or CD8(+) T-cells was reported (92).

Conclusively these findings strongly argue for new therapeutic approaches including combinations of the anti-angiogenic treatments with immunotherapies in addition to the current standard regimens for cancers, particularly for those that do not respond to surgery, chemotherapy, or radiation.

## Tumor endothelium mediated immunological consequences in the context of radiotherapy

Tumor eradication or local cancer control for a better outcome are the main goals of radiation therapy. Endothelial cells act as critical determinants of the radiation response in tumors as radiotherapy generally fosters endothelial apoptosis, increased vascular permeability, and acquisition of a pro-inflammatory and -coagulant phenotype (93–95). The radiation sensitivity of vessels in general correlates with their morphology: capillaries and small vessels (like angiogenic tumor vessels) are extremely sensitive to ionizing radiation, whereas larger blood vessels seem to be less affected (96, 97). Radiation induces phenotypic changes of tumor endothelial cells (e.g., apoptosis or senescence) as well as wide range of microenvironmental changes by production and secretion of reactive oxygen and nitrogen species, growth and chemotactic factors, which in turn govern recruitment of immune cells (11, 98, 99).

As an apparent approach, sensitizing tumor endothelial cells to radiation-induced apoptosis resulted in a more pronounced tumor growth delay upon irradiations in preclinical animal models, which suggested that a therapeutic targeting at the level of the tumor vasculature could counteract radiation resistance (97, 100). In contrast, in an elegant preclinical study Moding et al. reported that radiosensitizing endothelial cells did not increase local tumor control of soft tissue sarcomas after stereotactic body radiation therapy (101). Furthermore, proangiogenic factors including VEGF can rapidly repress radiation induced ceramide generation, and subsequently endothelial apoptosis (102). Therefore, targeting endothelial cells aiming at achieving complete tumor starvation is not supposed to be curative. More likely, approaches that improve the vascular function and thus tumor oxygenation as well as the recruitment and activation of immune cells by tumor endothelial cells gained attraction also in radiation therapy to enhance the sensitivity of the tumors to ionizing radiation.

To improve blood perfusion and thus tumor oxygenation, again vascular normalization using anti-angiogenic-therapy was suggested (103, 104). According to this hypothesis Koo et al. recently showed that a combined radiotherapy and anti-angiogenic treatment (with the second-generation multi-targeted receptor tyrosine kinase inhibitor sunitinib malate, which inhibits PDGF and VEGF) showed synergistic effects in anti-cancer treatment using heterotopic human lung cancer xenografts (105). Herein, radiation induced extensive necrosis in the central portion of the tumors, as the immature tumor blood vessels were sensitive to radiotherapy. The resulting decreased vascular supply created then a hypoxic area and decreased the tumoricidal effect of radiotherapy by reducing the oxygen-free radicals. When radiotherapy was then combined with anti-angiogenic treatment that inhibits the formation of immature blood vessels, the tumor perfusion was maintained and tumor necrosis was reduced. This treatment combination resulted then in a more significantly suppressed tumor growth, as vessel normalization, which achieved an efficient tumor perfusion, significantly improved tumor oxygenation that is prerequisite for the tumoricidal effects of ionizing radiation (105). In line with these findings Zhu et al. could show that inhibition of hypoxia-induced angiogenesis limits the efficiency of radiotherapy (106). Radiotherapy-sensitive lung tumors were characterized by low levels of hypoxia inducible factor-1α and VEGF, which may reflect better oxygenated tumors with less angiogenic and thus more matured blood vessels. In contrast, high expression levels of the respective genes were detected in radiotherapy-resistant lung tumors which might be based on the hypoxic tumor microenvironment with more angiogenic tumor blood vessels (106). Conclusively, combining anti-angiogenic treatment with radiation therapy can achieve better tumor control as oxygen is a potent radiosensitizer; this may result in the use of lower radiation doses, as thus minimizing treatment-related normal tissue toxicity (107).

In general, the pharmacological inhibition of pro-angiogenic factors triggers apoptosis of angiogenic endothelial cells in the immature and leaky tumor blood vessels leading to the selection for mature, non-leaky vessels, the so-called pruning effect. Within these matured and normal vessels endothelial anergy is restored. Jaillet et al. reported that ionizing radiation altered the glycosylation pattern of endothelial cells, in particular increased high mannose-type N-glycans and decreased glycosaminoglycans, which stimulated the interactions between irradiated endothelial cells and monocytes (108). Thus, targeting either the endothelium glycome may be considered as therapeutic target for modulating the inflammatory response or combining radiation therapy that seems to reduce endothelial anergy with anti-immunosuppressive therapy. Indeed, re-activation of the tumor vasculature was also shown to improve the therapeutic outcome of radiotherapy combined with immune-modulators. e.g., vessel specific-delivery of IL2, a cytokine known to stimulate the proliferation of cytotoxic T-cells, natural killer cells, and regulatory T-cells, resulted in an additive or synergistic anti-tumor effect when the administration of this immunocytokine was combined with radiotherapy (109). Tumor endothelium specific targeting was achieved by coupling IL2 to the small immune protein L19 that recognizes the extra domain B (ED-B) of fibronectin associated with tumor neovasculature. Of note, specifically addressing the tumor vasculature resulted in higher and thus more effective intratumoral local concentration of IL2 while reducing side effects, as the high doses used by systemic administration to reach an effective intratumoral dose of IL2 often leads to toxicity (e.g., capillary leakage) (109, 110).

In addition, preclinical and clinical evidence exists for the immuno-stimulatory properties of radiotherapy. Radiation treatment can foster immunogenic tumor cell death whereby danger-associated molecular patterns (DAMPs, e.g., calreticulin and adenosine triphosphate) were released which in turn can recruit and activate dendritic cells to process tumor antigens for naïve T-cells finally resulting in an anti-tumor immune responses (111, 112). Of note, radiation-induced tumor-targeted immunotherapy was shown to improve the therapeutic index and to extend the reach of immunomodulatory agents (113). In particular, radiation induced upregulation of VEGF expression was used to target 4-1BB/ CD137, a major immune-stimulatory receptor expressed on activated CD8(+) T-cells, to the irradiated tumor as well as to distant tumor lesions. This innovative method used radiation therapy to extend tumor-targeted immunotherapy also to VEGF low tumors. Radiation-induced tissue injury, which is known to trigger angiogenic processes, is accompanied by upregulation of VEGF expression, especially in lesions expressing low levels of VEGF. The agonistic 4-1BB oligonucleotide aptamer was conjugated to an aptamer that binds to VEGF (114). The administration of this conjugate after tumor irradiation was used to induce an optimal 4-1BB co-stimulation at the tumor site that in turn enhanced tumor immunity and inhibited tumor growth, while no toxicities classically associated with systemic administration of 4-1BB ligands was observed. Thus, systemically administered but specifically tumor-/ VEGF-targeted 4-1BB co-stimulation in combination with radiation elicited a potent antitumor immune response capable of controlling the growth of distant non treated subcutaneous and metastatic breast tumor lesions (113). This anti-tumor T-cell activation as a result from tumor-localized radiation-induced anti-tumor immune responses strongly argues for a synergistic effect of radiotherapy with immune checkpoint inhibitors (115).

## Conclusion

The tumor vascular endothelium is a key cell compartment for the response of tumors to cancer therapy. The tumor initiated neovascularization for nutrients and oxygen supply prior tumor progression results in a structural and functional abnormal tumor vasculature, which contributes to a pro-tumorigenic and immunosuppressive environment altering the therapy response of tumor cells. In particular for clinically approved immunotherapies, such as immune checkpoint blockade and adoptive T-cell transfer, the functional abnormal tumor vasculature fosters therapy resistance by limiting an inefficient recruitment, distribution and infiltration of tumor eradicating immune cells. Therefore, tumor vasculature targeting agents in order to re-activate specifically the tumor endothelial cells in terms of vascular normalization provide promising strategies to optimize the efficacy of currently employed cancer therapies, especially immunotherapies.

## Author contributions

The author confirms being the sole contributor of this work and approved it for publication.

### Conflict of interest statement

The author declares that the research was conducted in the absence of any commercial or financial relationships that could be construed as a potential conflict of interest.
